# Data-based surrogate modeling and performance prediction of MEMS touch mode capacitive sensing using deep multilayer perceptron networks

**DOI:** 10.3389/frai.2026.1960066

**Published:** 2026-10-19

**Authors:** Pushparaj E, Sumit Kumar Jindal

**Affiliations:** School of Electronics Engineering, Vellore Institute of Technology, Vellore, Tamil Nadu, India

**Keywords:** five-fold cross-validation, deep neural network, MEMS capacitive pressure sensor, multilayer perceptron, surrogate model

## Abstract

**Introduction:**

This work presents a structurally optimized rectangular diaphragm that operates in touch mode as the basis for a high-performance micro-electro-mechanical system (MEMS) capacitive pressure sensor (CPS). Rectangular diaphragms offer significant advantages in terms of structural control, constant stress handling and directional extension of the contact region.

**Methods:**

The proposed framework encompasses structural design, analytical modeling, numerical computation, finite element analysis (FEA) and Artificial Intelligence (AI)-based prediction. In this design, greater directional control results in a smoother and more reliable change in capacitance with pressure. The rectangular diaphragm design can be changed to smooth the transition between normal and touch modes and produce more consistent linearity and sensitivity. Numerical computation, FEA simulation and deep neural network (DNN) surrogate model for predicting the performance characteristics of a novel MEMS capacitive pressure sensor. A comprehensive dataset is constructed by combining Numerical computation and FEA-simulated results across a wide range of diaphragm geometries and applied pressure conditions. A DNN surrogate model is subsequently developed and trained on this dataset to predict the performance characteristics of the proposed MEMS capacitive pressure sensor.

**Results:**

The model achieves an overall mean absolute percentage error (MAPE) of 0.22 % and R2 above 0.999 across all four outputs, a substantial speed improvement, with prediction time reduced to a matter of seconds compared to the tens of min required for direct FEA simulation.

**Discussion:**

The results show that the proposed rectangular diaphragm effectively controls the touch region. It also improves capacitance and sensitivity. The DNN surrogate model predicts sensor performance precisely and efficiently. It enables rapid design-space exploration for MEMS capacitive pressure sensors.

## 1 Introduction

Throughout the past four decades, capacitive and piezo resistive micro-machined pressure sensors have been among the most successful MEMS to be invented. The use of pressure sensors in high performance applications includes power plants, automobiles, industrial, biomedical, and environmental monitoring. Using a CPS allows for high pressure sensitivity, minimal temperature drift on and off, low noise and reduced dependence on side stress (Belwanshi, 2021). Deflection, capacitance and sensitivity in CPS of a clamped-edge circular are assessed using a comprehensive mathematical approach in both the Un-touch and touch modes (Ko and Wang, 1999). A mathematical model of double touch mode CPS enables precise capacitance and sensitivity estimations. Simulation forecasting supports design optimization and validates performance gains. The effects of a double-sided diaphragm on capacitance and sensitivity in touch mode CPS are examined using a numerical model (Lv et al., 2008; Kang et al., 2017). A robust MEMS touch-mode CPS with a flexible SiC circular diaphragm is shown. In order to compare and optimize performance, mathematical approach and simulation are employed. MEMS touch mode CPS evaluation in detail using numerical simulation and mathematical approach with SiC film as the principle sensing element (Han et al., 2023). To improve the functionality of a touch-mode CPS, a novel design method is suggested. Numerical simulation and mathematical approach show increased operational dependability and sensitivity (He et al., 2024). The capacitance response of a MEMS double touch mode CPS with a conical notch substrate is examined. Comparative performance analysis, numerical simulation and mathematical approach all promote a strong design framework (Shao et al., 2023). The capacitance response in a concave well substrate touch-mode CPS is analyzes mathematically and simulated. The findings direct performance optimization and provide insights into sensor behavior (Kang et al., 2021). MEMS CPS with square diaphragms are thoroughly analyses and their ideal design parameters are estimated using an effective analytical method. Enhancing model accuracy and optimizing performance are two benefits of the effort (Patel et al., 2024). Although capacitive pressure sensors have great sensitivity and precision, they have hysteresis and nonlinearity. In order to minimize contact area and minimize hysteresis, the suggested line-touch mode construction incorporates a recessed lower electrode; In contrast to conventional touch mode structures, greatly increases linearity and the linear response range of the sensor (Chuai et al., 2024). A MEMS-based MIM (Metal–Insulator–Metal) CPS featuring a gold-silicon nitride structure tailored for hostile environments is designed. The sensor's great sensitivity and linear response over a broad pressure range are demonstrated by its design and simulation in FEA (Srinivasa Rao et al., 2020). Through structural optimization utilizing the Taguchi technique, a silicon carbide capacitor pressure sensor featuring a aluminum nitride layer and vacuum cavity structure has been presented for harsh-environment applications, exhibiting minimal nonlinearity and increased sensitivity (Zhou et al., 2023). When compared to traditional diaphragm structures, a perforated circular diaphragm combined with crab-leg meandering designs has been shown to increase the sensitivity of MEMS capacitor pressure sensors in low pressure healthcare monitoring applications (Jagabathuni and Peravali, 2025). For low-pressure sensing tasks, a MIM-based MEMS capacitor pressure sensor with an layered structure and a flexion-supported square diaphragm has been disclosed, demonstrating enhanced mechanical and capacitive sensitivity (Jagabathuni et al., 2025). With a circular diaphragm and conical notch substrate to improve capacitance and sensitivity, In Ke et al. (2021), the author introduces a MEMS-based touch mode CPS through analytical modeling and Numerical computation and FEA simulations are verified, demonstrating enhanced performance above current designs. Using finite element simulations, the author examines the displacement and stress behavior of square, rectangular and circular diaphragms for micro pressure sensors. The study Li et al. (2022) helps choose the best diaphragm geometry for increased output range, linearity and sensitivity. A rectangular diaphragm-based MEMS-based piezo resistive pressure sensor is analytically modeled is discussed. To improve overall performance, the sensor makes use of improved diaphragm cut patterns. A thorough finite element study of a piezoresistive MEMS pressure sensor is conducted by the author (Abedi et al., 2025). So, this work introduces the rectangular diaphragm for its superior stress distribution, ease of integration into flexible electronic systems, higher fill factor in sensor arrays and enhanced control over the touch region particularly in double-touch mode applications. Physical modeling, comprising material property research and structural design, is the first step in the proposed work. Despite their widespread use, the design and optimization of MEMS capacitive pressure sensors remain computationally intensive. Multi-physics finite element analysis (FEA) tools (Mishra et al., 2012; Jakati et al., 2016; Lobur et al., 2016; Zhang et al., 2019) deliver high-fidelity predictions of deflection, stress and capacitance, but a single simulation requires approximately 45 min per parameter set. Exhaustive design-space exploration across even moderate-dimensional parameter grids is therefore prohibitively expensive for front-end engineering. Machine learning surrogate models provide a compelling alternative. Once trained offline on representative FEA simulation data, a DNN can predict sensor performance in seconds with negligible error, enabling rapid parametric sweeps and design optimization (Zhai et al., 2022; Niu and Wang, 2022; Park et al., 2021; Chen and Chen, 2022). Recent literature has demonstrated DNN surrogates for thermal battery optimization (Park et al., 2021) and two-output capacitive sensor prediction (Chen and Chen, 2022). However, existing MEMS-DNN studies commonly evaluate performance on a single random hold-out split, which can produce overly optimistic, high-variance and potentially non-reproducible performance estimates. This work addresses the identified limitations through three key contributions. First, a 3,000-sample FEA generated dataset is constructed covering seven input design features which incorporating material properties such as Young's modulus and Poisson's ratio that vary across real manufacturing scenarios, thereby enabling a more comprehensive representation of practical sensor design space. Second, an MLP architecture with four hidden layers of 20 neurons each is employed, extending four simultaneous output targets, namely membrane deflection, output capacitance, capacitive sensitivity and mechanical sensitivity (dw/dP), to provide a more complete characterization of sensor performance. Third, a rigorous evaluation protocol combining five-fold cross-validation on 90% of the data with a strictly sealed 10% hold-out test set is applied, ensuring statistically robust, reproducible and unbiased performance estimates that overcome the reliability limitations of the single random split approach used in prior studies.

The purpose of this work is to address that demand and further optimize the field of study. The model also confirms that the suggested structure performs better and is more reliable under differential pressure situations, which makes it appropriate for high-precision applications like aircraft transducers, power plants, automobiles, industrial, and etc. Efficient study of touch mode functioning is necessary; touch mode CPS performs better in many applications. Thus, the capacitance, sensitivity and dw/dP of a silicon-based CPS with Rectangular diaphragm in both untouch and conduct mode operation are thus thoroughly and analytically derived in this work. Simulation results are used to generate data in order to produce a dataset with enough variability. The data is then preprocessed and an DNN model is created, trained and verified as part of the AI/ML process.

## 2 Materials and methods

Figure 1 depicts the entire workflow of the proposed work. The CPS works using two parallel plates, where a flexible silicon diaphragm acts as one plate and a silicon substrate acts as the other. When pressure is applied, the diaphragm bends and reduces the gap between the plates, changing the capacitance. The sensor works in three stages: Normal Mode, Transition mode and touch mode full contact with the bottom insulating layer. As pressure increases and the diaphragm touches more bottom area, capacitance rises sharply improving sensitivity. A rectangular diaphragm is preferred over circular or square ones as it has a larger flat center, better shape control and directional flexibility giving more accurate pressure response. Silicon is selected for both the diaphragm and substrate due to its strong, lightweight and flexible nature. It bends reliably under pressure without permanent damage and is highly compatible with MEMS fabrication processes. The sensor geometry and material are carefully optimized to get a smooth and predictable capacitance response. Mathematical models and FEA simulations confirmed the superior performance of the rectangular silicon diaphragm. However, running simulations for every design variation is time consuming and computationally expensive. To overcome this, a DNN is trained on simulation data to quickly predict sensor performance without running new simulations. This combined approach of analytical modeling, FEA simulation and DNN makes sensor design faster, accurate and cost efficient.

**Figure 1:**
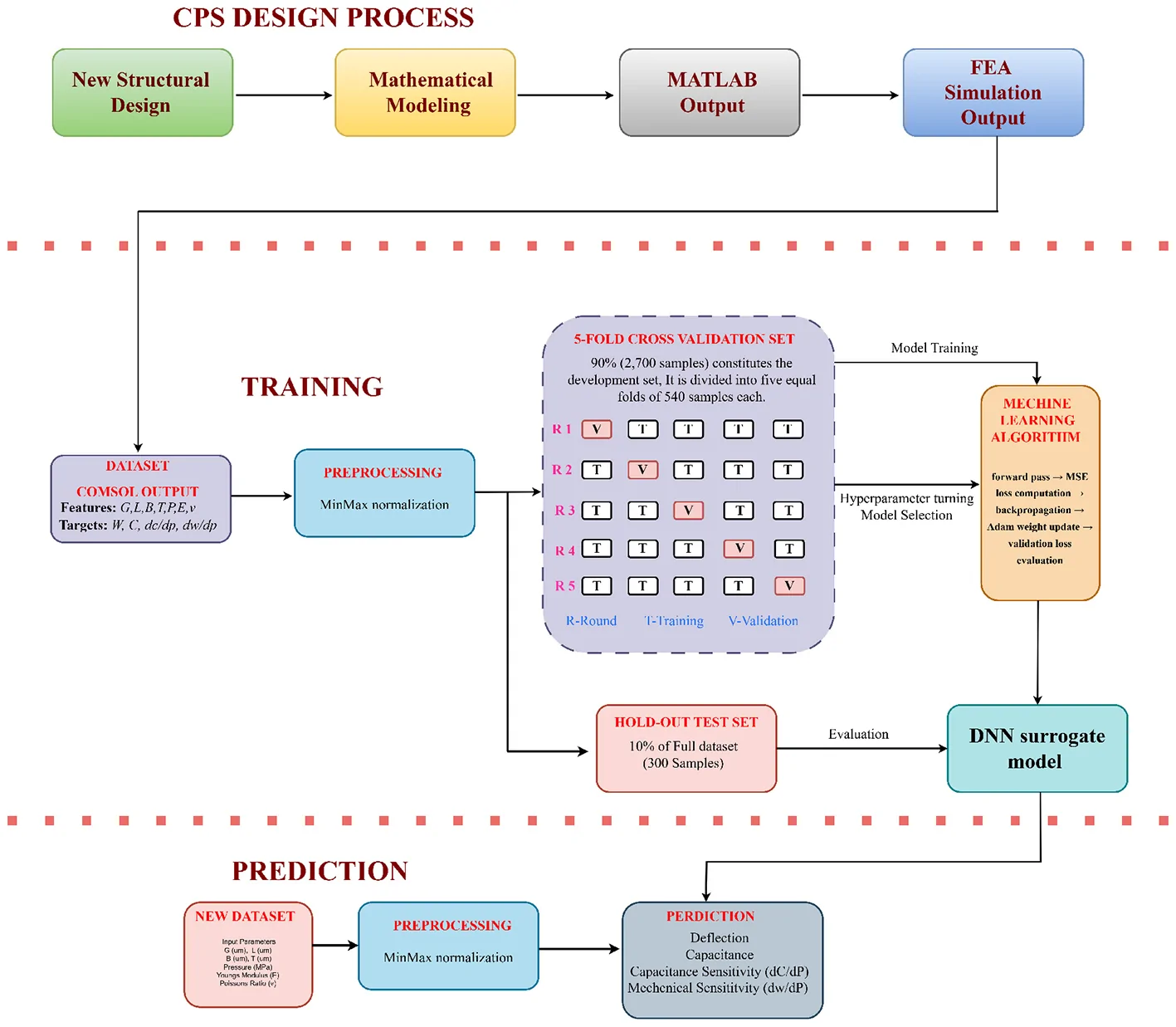
The block diagram shows the entire process of the proposed system.

## 3 The proposed CPS modeling specifications and operation principle

The idea of parallel plate capacitance is being used here, with substrate material acting as the second electrode and a diaphragm that is flexible acting as the first electrode. The diaphragm experiences mechanical displacement when pressure is applied, which causes a changing electrode gap that modifies the effective capacitance. The designed rectangular diaphragm sensor sectional representation is displayed in Figure 2. A near-linear capacitance to pressure response can be achieved across a specified working range by optimizing the diaphragm geometry, material characteristics and electrode arrangement as shown in Equation (1). A basic Capacitance equation:


$$\begin{array}{l} c=\frac{A\epsilon }{d} \end{array}$$ (1)

**Figure 2:**
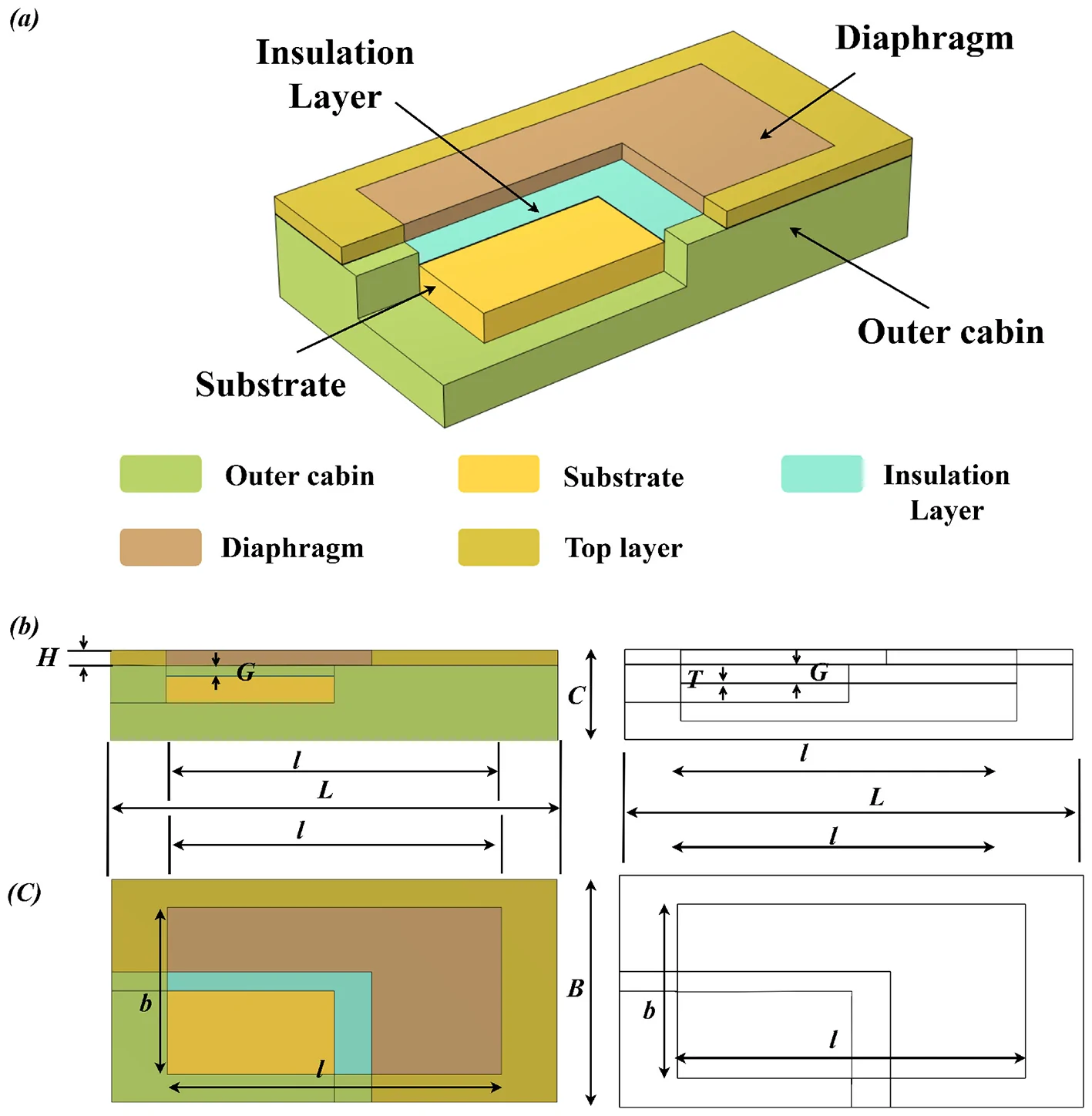
3D and cross-sectional views of the proposed rectangular diaphragm MEMS capacitive pressure sensor; **(a)** 3D view of the proposed sensor structure showing the diaphragm, insulation layer, outer cabin and substrate **(b)** cross-sectional view showing the diaphragm thickness *H*, initial gap *G*, structural dimensions (Length of Outer cabin L and Length of the Substrate *l*) and Thickness of the Insulation Layer *T* **(c)** top view showing the diaphragm length *l*, breadth of the substrate *b* and Breath of the Outer cabin B dimensions.

Normal mode, transition mode and touch mode are the three different ways that a CPS with a rectangular diaphragm operates. The normal mode is the area where the diaphragm deflects under pressure without touching the hollow floor. The applied pressure and capacitance develop a highly nonlinear relationship when pressure rises and the diaphragm makes contact with the cavity surface, this intermediate condition is known as the transition mode. The sensor switches to the touch mode when the diaphragm makes complete contact with the insulating layer at the cavity bottom. Two regimes can be distinguished for analytical modeling under the linear elastic approximation: normal mode and touch mode. The pressure capacitance behavior of the sensor can be accurately predicted by deriving unique equations for capacitance and deflection for each regime using plate theory and suitable boundary conditions for a rectangular diaphragm.

Figure 2 shows a cross sectional view of CPS. In Table 1 the design specifications are displayed. A thinner diaphragm may bend more when pressure is applied, it increases sensitivity. The diaphragm first bends in normal mode when pressure is applied and capacitance gradually rises. The diaphragm starts to come into contact with the bottom electrode at it's center as the pressure rises more and approaches the touch threshold. As the pressure increases, this center touch region spreads outward. Since capacitance is inversely related to gap and the gap distance is almost zero in this location, the capacitance increases with contact area increased. As a result, the overall capacitance increases dramatically as the touch region grows. This significant rise improves the sensor sensitivity. Regarding touch mode CPS, the rectangular diaphragm offers a number of benefits over the circular diaphragm, particularly with regard to sensitivity, capacitance and touch region management. How and why it can be improved is as follows: first, With a rectangular diaphragm, the touch region can be more precisely shaped by simply adjusting the length-to-width ratio, also known as the aspect ratio. As a result, the touch area expansion under pressure can be better controlled. Conversely, a circular diaphragm restricts geometric control; it always grows symmetrically from its center. Second, when compared to a circular diaphragm of the same area, the rectangular design offers a bigger flat middle area. An expanded touch region is created by a rectangular diaphragm under increased pressure, increasing the effective contact area and hence increasing the touch mode capacitance. Third, a rectangular diaphragm directional flexibility makes pressure induced deflection and touch region expansion more noticeable along its longer side. This makes it possible for capacitance to increase more sharply, improving capacitance sensitivity (dC/dP) in touch mode. Finally, compared to a circular diaphragm, a rectangular diaphragm provides superior control over the contact zone in touch mode, leading to larger capacitance, improved sensitivity and more design flexibility. This is particularly advantageous for flexible electronics and MEMS CPS design.

**Table 1:** Design parameters of the rectangular diaphragm.

Symbol	Parameter	Units	Design range/value
*P*	Applied pressure	MPa	0–2
*E*	Young's modulus	GPa	140–190 (nominal: 170)
*h*	Diaphragm thickness	μ*m*	10–30 (nominal: 20)
L	Diaphragm length	μ*m*	600–1200 (nominal: 900)
B	Diaphragm breadth	μ*m*	300–600 (nominal: 450)
ν	Poisson's ratio for silicon	–	0.17–0.28 (nominal: 0.28)
ε_*i*_	Dielectric constant of SiO_2_	–	3.7
ε_0_	Dielectric constant of air	–	1
*d*_0_/*G*	Initial gap depth	μ*m*	1–3 (nominal: 2)
*t*	Insulation layer thickness	μ*m*	0.5

## 4 Mathematical approach for the proposed CPS design

According to thin plate theory, the fundamental deflection equation for a thin, isotropic rectangular plate under transverse force is obtained for simply supported, clamped rectangular diaphragm under uniform pressure, the governing equation for small deflections is:


$$\begin{array}{l} \nabla^{4}w=\frac{P}{D} \end{array}$$ (2)

where: ∇^4^ is the bi-harmonic operator (fourth-order differential operator): The governing equation and biharmonic operator for the diaphragm under uniform pressure are given by Equations (2) and (3).


$$\begin{array}{l} \nabla^{4}w=\frac{\partial^{4}w}{\partial x^{4}}+2\frac{\partial^{4}w}{\partial x^{2}\partial y^{2}}+\frac{\partial^{4}w}{\partial y^{4}} \end{array}$$ (3)

The boundary conditions for simply supported, clamped edges length and breath are 2*l* and 2*b*. The diaphragm deformation, *w*(*x, y*), depends on the *x* and *y* directions, which are provided by Timoshenko and Woinowsky-Krieger (1959).


$$\begin{array}{l} w\left(x,y\right)=\frac{P}{24D}\frac{{\left(l^{2}-x^{2}\right)}^{2}{\left(b^{2}-y^{2}\right)}^{2}}{l^{4}+b^{4}} \end{array}$$ (4)

Flexural rigidity of the diaphragm and the deflection distribution are given by Equations (4) and (5):


$$\begin{array}{l} D=\frac{Eh^{3}}{12\left(1-\nu^{2}\right)} \end{array}$$ (5)

where:

- Young's modulus (*E*).
- The diaphragm thickness (*h*).
- Poisson's ratio (ν).

### 4.1 Maximum deflection of rectangular diaphragm under uniform pressure


$$\begin{array}{l} w_{0}=w\left(0,0\right)=\frac{P}{24D}\frac{l^{4}b^{4}}{l^{4}+b^{4}} \end{array}$$ (6)

where:

- *P* is the applied pressure.
- *l* and *b* are the half-lengths of the diaphragm in the *x* and *y* directions, respectively. The maximum central deflection and deflection profile are given by Equations (6) and (7).

Deflection profile


$$\begin{array}{l} w\left(x,y\right)=w_{0}{\left(1-\frac{x^{2}}{l^{2}}\right)}^{2}{\left(1-\frac{y^{2}}{b^{2}}\right)}^{2} \end{array}$$ (7)

Variable definition and assumption are given by (8)–(10).


$$\begin{array}{ll} u=l^{2}-x^{2}, & x=\sqrt{l^{2}-u} \end{array}$$ (8)

$$\begin{array}{ll} v=b^{2}-y^{2}, & y=\sqrt{b^{2}-v} \end{array}$$ (9)

Assumption


$$\begin{array}{l} A{\left(l^{2}-x^{2}\right)}^{2}{\left(b^{2}-y^{2}\right)}^{2}\ll d_{0} \end{array}$$ (10)

### 4.2 Binomial expansion


$$\begin{array}{l} \frac{1}{d_{0}-\varepsilon }=\frac{1}{d_{0}}+\frac{\varepsilon }{d_{0}^{2}}+\cdots \end{array}$$ (11)

$$\begin{array}{l} \frac{1}{d_{0}-Af\left(x,y\right)}=\frac{1}{d_{0}}+\frac{Af\left(x,y\right)}{d_{0}^{2}} \end{array}$$ (12)

where the binomial expansion and its use in the capacitance formulation are given by (11)–(13).


$$\begin{array}{l} f\left(x,y\right)={\left(l^{2}-x^{2}\right)}^{2}{\left(b^{2}-y^{2}\right)}^{2} \end{array}$$ (13)

### 4.3 Calculating the normal mode capacitance

The normal-mode capacitance and its integration terms are given by (14)–(21).


$$\begin{array}{l} C=\varepsilon_{0}\int_{-l}^{l}\int_{-b}^{b}\frac{1}{d_{0}-A{\left(l^{2}-x^{2}\right)}^{2}{\left(b^{2}-y^{2}\right)}^{2}}dxdy \end{array}$$ (14)

$$\begin{array}{l} C=\varepsilon_{0}\int_{-l}^{l}\int_{-b}^{b}\left[\frac{1}{d_{0}}+\frac{A{\left(l^{2}-x^{2}\right)}^{2}{\left(b^{2}-y^{2}\right)}^{2}}{d_{0}^{2}}\right]dxdy \end{array}$$ (15)

$$\begin{array}{l} C=\frac{\varepsilon_{0}}{d_{0}}\int_{-l}^{l}\int_{-b}^{b}dxdy\\ \;+\frac{\varepsilon_{0}A}{d_{0}^{2}}\int_{-l}^{l}\int_{-b}^{b}{\left(l^{2}-x^{2}\right)}^{2}{\left(b^{2}-y^{2}\right)}^{2}dxdy \end{array}$$ (16)

Integration of the first term


$$\begin{array}{l} \int_{-l}^{l}\int_{-b}^{b}dxdy=4lb \end{array}$$ (17)

Integration of the second term


$$\begin{array}{l} \int_{-l}^{l}{\left(l^{2}-x^{2}\right)}^{2}dx=I_{x} \end{array}$$ (18)

$$\begin{array}{l} \int_{-b}^{b}{\left(b^{2}-y^{2}\right)}^{2}dy=I_{y} \end{array}$$ (19)

$$\begin{array}{l} \int_{-l}^{l}\int_{-b}^{b}{\left(l^{2}-x^{2}\right)}^{2}{\left(b^{2}-y^{2}\right)}^{2}dxdy=I_{x}I_{y} \end{array}$$ (20)

Final normal-mode capacitance equation


$$\begin{array}{l} C=\frac{4lb\varepsilon_{0}}{d_{0}}\left[1+\frac{64}{225}\frac{P}{P_{t}}\right] \end{array}$$ (21)

### 4.4 Evaluation of touch-mode capacitance (Ct) using integral approximation

The touch-mode capacitance is given by (22)–(28).


$$\begin{array}{l} C_{t}=\frac{\varepsilon_{0}\sin\theta }{\theta d_{0}}\int_{-\frac{3l}{4}}^{\frac{3l}{4}}\int_{-\frac{3b}{4}}^{\frac{3b}{4}}dxdy\\ \;+\frac{\varepsilon_{0}\sin\theta }{\theta d_{0}^{2}}\cdot \frac{Pl^{2}b^{2}}{24D}\int_{-\frac{3l}{4}}^{\frac{3l}{4}}\int_{-\frac{3b}{4}}^{\frac{3b}{4}}{\left(1-\frac{x^{2}}{l^{2}}\right)}^{2}{\left(1-\frac{y^{2}}{b^{2}}\right)}^{2}dxdy \end{array}$$ (22)

Integrating the first part


$$\begin{array}{l} \int_{-\frac{3l}{4}}^{\frac{3l}{4}}\int_{-\frac{3b}{4}}^{\frac{3b}{4}}dxdy=\left(\frac{3l}{2}\right)\left(\frac{3b}{2}\right)=\frac{9lb}{4} \end{array}$$ (23)

Integrating the second part


$$\begin{array}{l} \int_{-\frac{3l}{4}}^{\frac{3l}{4}}\int_{-\frac{3b}{4}}^{\frac{3b}{4}}{\left(1-\frac{x^{2}}{l^{2}}\right)}^{2}{\left(1-\frac{y^{2}}{b^{2}}\right)}^{2}dxdy=J_{x}J_{y} \end{array}$$ (24)

Where


$$\begin{array}{l} J_{x}=\int_{-\frac{3l}{4}}^{\frac{3l}{4}}{\left(1-\frac{x^{2}}{l^{2}}\right)}^{2}dx \end{array}$$ (25)

$$\begin{array}{l} J_{y}=\int_{-\frac{3b}{4}}^{\frac{3b}{4}}{\left(1-\frac{y^{2}}{b^{2}}\right)}^{2}dy \end{array}$$ (26)

Final touch mode capacitance


$$\begin{array}{l} C_{t}=\frac{\varepsilon_{0}\sin\theta }{\theta d_{0}}\left(\frac{9lb}{4}\right)+\frac{\varepsilon_{0}\sin\theta }{\theta d_{0}^{2}}\frac{Pl^{3}b^{3}}{24D}\left(1.066\right) \end{array}$$ (27)

$$\begin{array}{l} C_{t}=\frac{\varepsilon_{0}\sin\theta }{\theta }\left[\frac{9lb}{4d_{0}}+\frac{1.066Pl^{3}b^{3}}{24Dd_{0}^{2}}\right] \end{array}$$ (28)

### 4.5 Evaluation of untouch-mode capacitance (*C*_*ut*_) using integral approximation

The untouched-mode and total capacitance are given by (29)–(32)


$$\begin{array}{l} C_{ut}=\frac{\varepsilon_{0}\sin\theta }{\theta d_{0}}\iint dxdy\\ \;+\frac{\varepsilon_{0}\sin\theta }{\theta d_{0}^{2}}\frac{Pl^{2}b^{2}}{24D}\iint {\left(1-\frac{x^{2}}{l^{2}}\right)}^{2}{\left(1-\frac{y^{2}}{b^{2}}\right)}^{2}dxdy \end{array}$$ (29)

$$\begin{array}{l} x\in \left[\frac{3l}{4},l\right],\;y\in \left[\frac{3b}{4},b\right] \end{array}$$ (30)

Final UN-touch capacitance


$$\begin{array}{l} C_{ut}=\frac{\varepsilon_{0}\sin\theta }{\theta d_{0}}lb\left[\frac{1}{16}+\frac{Plb}{24Dd_{0}}\left(1.432\right)\right] \end{array}$$ (31)

Total capacitance (touch + untouch regions)


$$\begin{array}{l} C_{\text{total}}=\frac{\varepsilon_{0}\sin\theta lb}{\theta d_{0}}\left[\frac{13}{64}+\frac{1.066Pl^{2}b^{2}}{24Dd_{0}}+\frac{1.432Plb}{24D}\right] \end{array}$$ (32)

### 4.6 Solution to determine capacitive sensitivity (dC/dP)

Capacitive sensitivity is the change in capacitance for a change in pressure. Capacitive sensitivity is therefore provided by

Differentiating *C* with respect to *P* as shown in the (33) – (38):


$$\begin{array}{l} \frac{dC}{dP}=C_{0}\frac{256}{225}\frac{1}{P_{t}} \end{array}$$ (33)

Since *C*_0_ is constant and *P*_*t*_ depends only on *l, b*, and material constants, this simplifies to:


$$\begin{array}{l} \frac{dC}{dP}=\frac{C_{0}}{P_{t}}\left(\frac{256}{225}\right) \end{array}$$ (34)

Substituting *C*_0_ and *P*_*t*_


$$\begin{array}{l} C_{0}=\frac{4lb\varepsilon_{0}}{d_{0}} \end{array}$$ (35)

$$\begin{array}{l} P_{t}=\frac{24D\left(l^{4}+b^{4}\right)d_{0}}{l^{4}b^{4}} \end{array}$$ (36)

$$\begin{array}{l} \frac{dC}{dP}=\left(\frac{\frac{4lb\varepsilon_{0}}{d_{0}}}{24D\left(l^{4}+b^{4}\right)d_{0}l^{4}b^{4}}\right)\left(\frac{256}{225}\right)\\ \;=\frac{4lb\varepsilon_{0}l^{4}b^{4}}{24D\left(l^{4}+b^{4}\right)d_{0}^{2}}\left(\frac{256}{225}\right) \end{array}$$ (37)

Simplifying the expression:


$$\begin{array}{l} \frac{dC}{dP}=\frac{256}{225}\frac{\varepsilon_{0}l^{5}b^{5}}{6D\left(l^{4}+b^{4}\right)d_{0}^{2}} \end{array}$$ (38)

### 4.7 Mathematical evaluation of dw/dP

When a change in applied pressure occurs, the deflection changes, which is known as dw/dP as shown in the (39) – (42). Thus, dw/dP is provided by the deflection at any point (*x, y*) on a rectangular diaphragm under uniform pressure *P* in the Equation 3, At the center of the diaphragm (*x* = 0, *y* = 0), the maximum deflection is:


$$\begin{array}{l} w\left(0,0\right)=\frac{P}{24D}\frac{l^{4}b^{4}}{l^{4}+b^{4}} \end{array}$$ (39)

The rate at which deflection changes in relation to pressure is known as the dw/dP *S*_*m*_:


$$\begin{array}{l} S_{m}=\frac{\partial w}{\partial P}{|}_{x=0,y=0} \end{array}$$ (40)

Differentiating *w*(0,0) with respect to *P* gives:


$$\begin{array}{l} S_{m}=\frac{\partial }{\partial P}\left(\frac{P}{24D}\frac{l^{4}b^{4}}{l^{4}+b^{4}}\right)\\ \;=\frac{1}{24D}\frac{l^{4}b^{4}}{l^{4}+b^{4}} \end{array}$$ (41)

$$\begin{array}{l} S_{m}=\frac{1}{24D}\frac{l^{4}b^{4}}{l^{4}+b^{4}} \end{array}$$ (42)

## 5 Optimization of diaphragm dimensions under different boundary conditions for maximum capacitance and sensitivity

### 5.1 Evaluation of diaphragm size for rectangular CPS

The sensor operates in normal mode for pressures between 0 and 0.02 MPa and in touch mode for pressures between 0.02 and 2 MPa. In CPS, deflection happens when the diaphragm bends downward toward the bottom electrode due to external pressure. Pressure magnitude, diaphragm dimensions (length, width and thickness) material qualities (Young's modulus and Poisson's ratio) and boundary conditions are some of the variables that affect the amount of deflection. A diaphragm maximum deflection usually happens in the middle and it symmetrically declines in length and width.

When the diaphragm and electrode come into contact in touch mode, a flat expanding area with bending edges is created. Usually, the deflection profile has a biquadratic or parabolic shape. Capacitance progressively rises when the diaphragm bends prior to contact. Capacitance and sensitivity rise quickly after touch. By changing the diaphragm aspect ratio, sensitivity and linearity can be adjusted.

The mechanical characteristics of rectangular diaphragms with different sizes under applied pressure is shown in detail in Figure 3. For analysis, it integrates both simulation data and diagrammatic representations. There are seven sub-figures in the Figures 3a–g. A particular facet of diaphragm shape and deformation characteristics is highlighted in each sub-figure. The sizes that are used are 1, 200 × 600 μm, 1, 000 × 500 μm, 900 × 450 μm, 800 × 400 μm, 600 × 300 μm. The length and width of five individual rectangular diaphragms, expressed in micrometers (μm) are shown in Figure 3a. For ease of identification, the diaphragms are layered and color-coded. Geometric scaling is seen in the figure, which consistently reduces size from greatest to smallest. Understanding dimensional fluctuations is facilitated by this depiction. When examining how size affects dw/dP and deflection, this variance is crucial.

**Figure 3:**
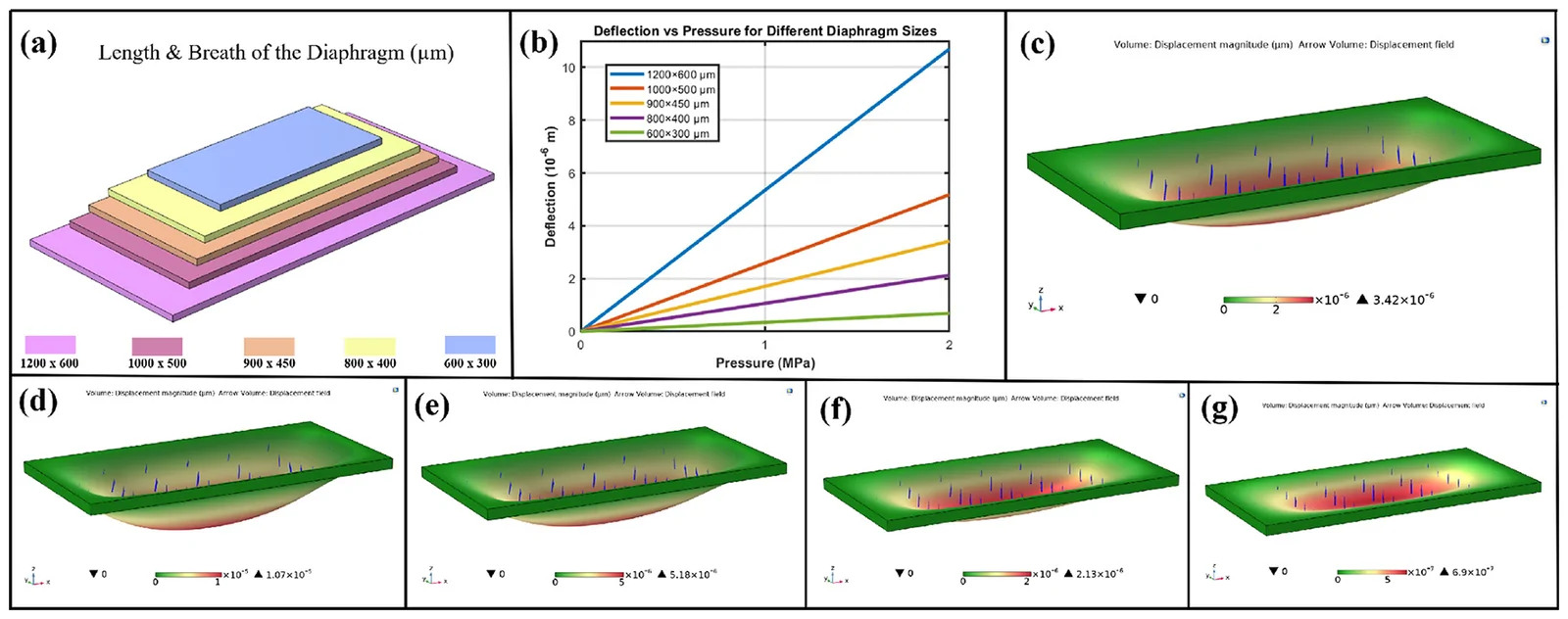
Geometrical configurations, pressure-deflection properties and simulated deformation profiles of rectangular MEMS diaphragms with varied lengths and widths; **(a)** rectangular diaphragms with different dimensions shown schematically, **(b)** for various diaphragm dimensions, the center diaphragm deflection varies with applied pressure, **(c)** rectangular diaphragm deformation field and three-dimensional displacement magnitude under uniform pressure loading for the proposed dimension 900 × 450μ*m*, **(d)** the 1, 200 × 600μ*m* diaphragm's simulated deformation variation, **(e)** the 1, 000 × 500μ*m* diaphragm's simulated deformation variation, **(f)** the 800 × 400μ*m* diaphragm's simulated deformation variation, **(g)** the 600 × 300μ*m* diaphragm's simulated deformation variation.

A graph of deflection vs. applied pressure for the various diaphragm diameters is shown in Figure 3b. The horizontal axis shows pressure in megapascals (MPa) whereas the vertical axis shows deflection in micrometers (× 10^−6^m). Each curve depicts a distinct diaphragm size and demonstrates elastic behavior by demonstrating a linear connection between pressure and deflection. The greatest deflection is displayed by the largest diaphragm 1, 200 × 600 μm and the least by the smallest 600 × 300 μm. Larger diaphragms are more sensitive to pressure, according to this tendency. With arrows indicating direction and intensity, the 3D simulation uses a color contour ranging from green (min) to red (max) to depict displacement magnitude. Typical of clamped rectangular diaphragms, maximum deflection happens at the center and progressively decreases toward the fixed edges.

3D displacement graphs for diaphragms of various diameters are shown in Figures 3d–g. Every figure illustrates how diaphragm size affect deformation. Higher central deflection and displacement values are seen in larger diaphragms Figures 3d, e. The displacement and deformation of smaller diaphragms are lower Figures 3f, g. These findings show that diaphragm size and stiffness are inversely related.

In every simulation, the displacement pattern is symmetrical. The center experiences the greatest deformation, while the edges show the least amount of movement. This validates boundary conditions that are effectively clamped. The impact of diaphragm sizes on deflection under applied pressure is clearly depicted in the illustration. Finite element simulation data, analytical graphs and geometric configurations are all integrated. All things considered, it provides a solid understanding of how small differences impact sensitivity and deformation behavior.

### 5.2 Evaluation of diaphragm thickness for rectangular CPS

Figure 4 illustrates how diaphragm thickness affects the deflection characteristics of a MEMS capacitive pressure sensor. Diaphragm structures with thicknesses of 30 μm, 25 μm, 20 μm, 15 μm, and 10 μm are depicted in Figure 4a using various colors. To investigate how changes in thickness impact the diaphragm's response under pressure, these structures Figures 4c–g are examined. The deflection increases linearly with applied pressure, according to the pressure-deflection graph (Figure 4b). The deflection of thicker diaphragms is lower than that of thinner ones. As thickness declines, sensitivity rises; the 10 μm diaphragm exhibits greatest deflection, while the 30 μm diaphragm exhibits minimum. Color contours and arrows that indicate direction and magnitude are used in 3D simulation results to show displacement dispersion.

**Figure 4:**
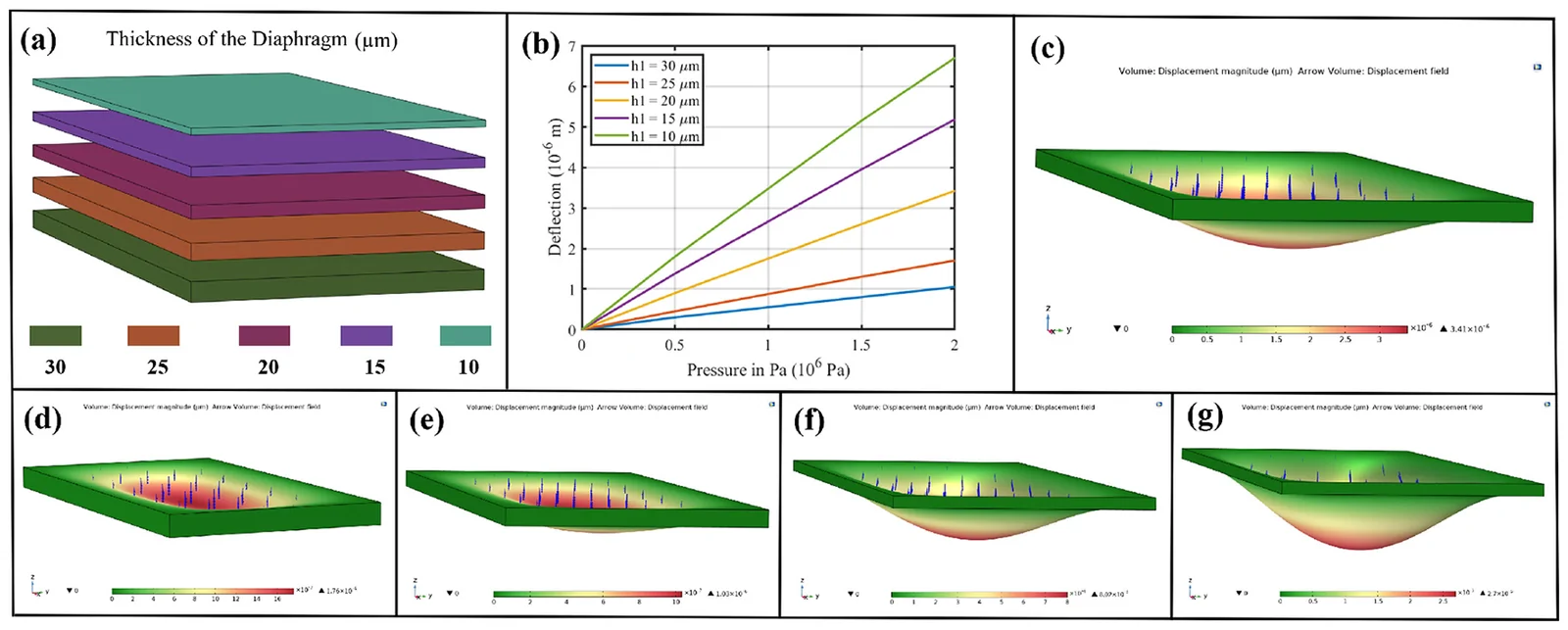
The impact of diaphragm thickness on the dw/dP of the MEMS capacitive pressure sensor; **(a)** schematic illustration of diaphragms with varied thicknesses (30 μ*m*, 25 μ*m*, 20 μ*m*, 15 μ*m*, and 10 μ*m*), **(b)** diaphragm deflection fluctuation for various thicknesses as a function of applied pressure, **(c–g)** three-dimensional displacement propagation and deformation profiles of the diaphragm against applied pressure for thicknesses of **(c)** 20 μ*m*, **(d)** 30 μ*m*, **(e)** 25 μ*m*, **(f)** 15 μ*m*, and **(g)** 10 μ*m*, exhibiting the rise in deflection with getting thinner thickness.

For thicker diaphragms, such 30 μm, the deformation is insignificant, suggesting increased structural rigidity and decreased sensitivity. As the thickness decreases from 25 μm to 10 μm, the central deflection gradually increases and a noticeable red patch that indicates maximum displacement develops in the middle. The thinnest diaphragm (10 μm) exhibits the largest deformation, indicating maximal sensitivity but less mechanical robustness. The Figure 4 illustrates the trade-off between sensitivity and structural dependability in MEMS pressure sensor design by showing that decreasing diaphragm thickness increases pressure sensitivity by increasing deflection, but it also results in decreased mechanical strength.

## 6 Diaphragm deflection analysis under applied pressure using Numerical computation

### 6.1 Deflection analysis of rectangular diaphragm in normal mode CPS

For normal mode operation, Figure 5a shows the capacitance variation with applied pressure. Within the pressure range of 0–0.02 MPa, it is evident that the capacitance varies linearly. This mode has a maximum capacitance variation of 4.4053 pF. During the transition from normal mode to touch mode, the bottom electrode is only just beginning to be touched by the sensing diaphragm. The transition region's pressure ranges from 0.02 to 0.5 MPa as mention in the Table 1.

**Figure 5:**
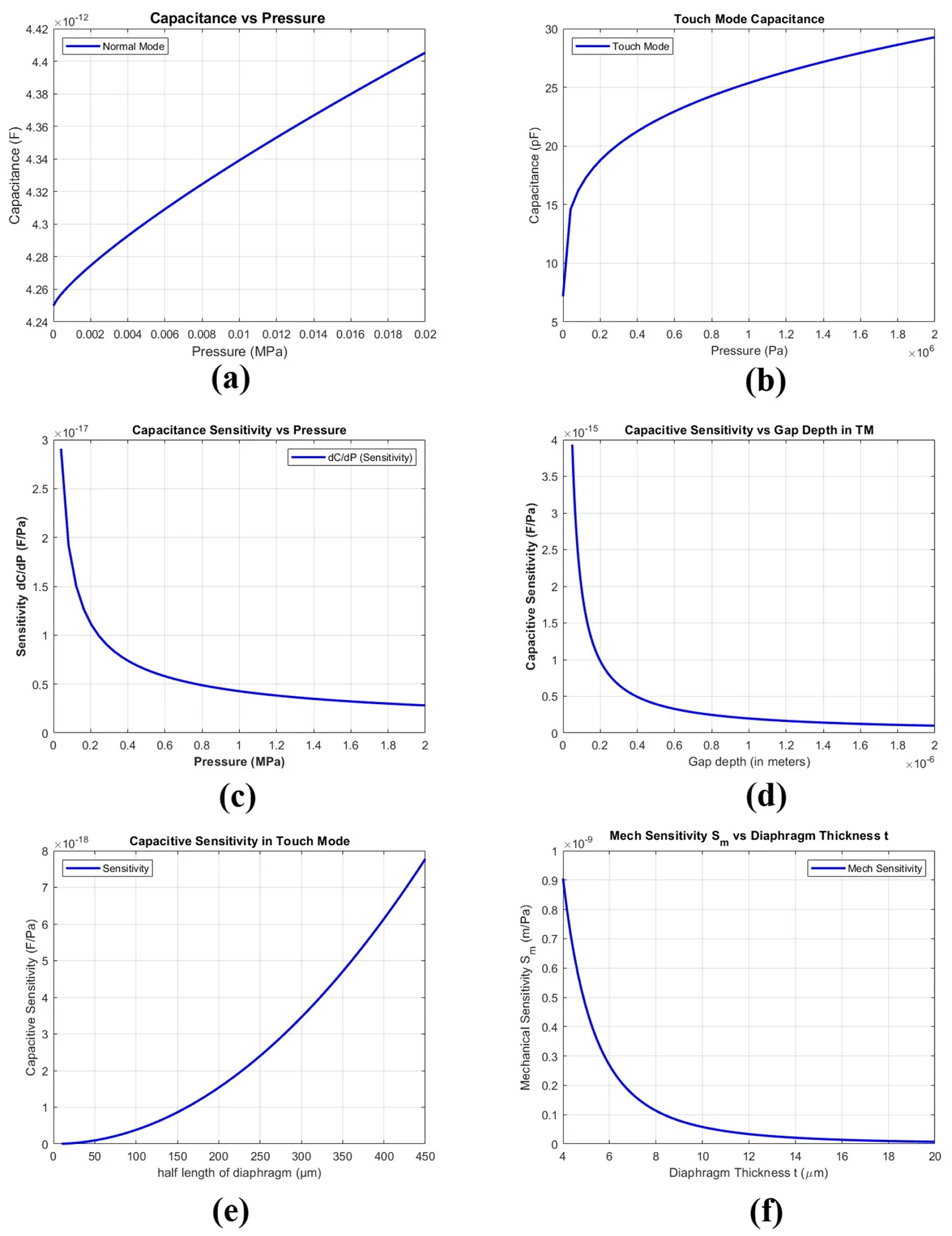
Numerical computation analysis of diaphragm deflection with applied pressure; **(a)** capacitive vs. pressure curve for normal mode capacitance, **(b)** capacitance vs. pressure curve of touch mode capacitance, **(c)** curve of capacitive sensitivity with applied pressure, **(d)** capacitive sensitivity vs. gap depth curve of proposed capacitive pressure sensors, **(e)** capacitive sensitivity with half length of diaphragm curve of capacitive pressure sensors, **(f)** curve of dw/dP for the proposed capacitive pressure sensors.

### 6.2 Deflection analysis of rectangular diaphragm in touch mode CPS

Figure 5b shows the diaphragm deflection variation for the specified pressure range, it is evident from the plot that the center deflection variation is linear. 0.5–2 MPa is the pressure range specified for this design Touch mode functioning as indicated in the above Table 1. This mode has the highest capacitance variation of 29.2688 pF at 2 Mpa pressure. We have already demonstrated the linear nature of the deflection variation for this pressure range.

### 6.3 Sensitivity analysis of the proposed rectangular CPS

In Figure 5c the capacitive sensitivity variation with diaphragm half-length is examined. When the diaphragm half-length surpasses 150 μ*m* under 0.5 MPa in touch mode, capacitive sensitivity dramatically rises. The sensitivity variation is negligible between 0 and 100 μ*m*. Thus, in order to reconcile high sensitivity and linearity, a half-length of 450 μ*m* is selected. The dC/dP change with gap-depth for touch mode operation is shown in Figure 5d. As shown in Figure 5e the capacitive sensitivity fluctuation with applied pressure in touch mode operation for the suggested rectangular diaphragm, exhibits enhanced linearity and controllability. Our design produces a linear capacitance-pressure connection over a wider range by achieving a more uniform diaphragm deflection. In particular, the sensor regulated center deflection results in a highly sensitive response in the beginning pressure range (0.02–0.5 MPa) with less nonlinearity than traditional designs. Up to 2 MPa, the linear operating range greatly increases, exhibiting steady and consistent sensitivity. For dependable sensing applications, this constant sensitivity is crucial. The suggested diaphragm maintains controllable sensitivity without early saturation, in contrast to traditional designs. This is accomplished by placing electrodes correctly and optimizing structural characteristics. Performance is enhanced by the rectangular diaphragm design, which also improves mechanical compliance and tunability. As a result, our work exhibits excellent linearity and adjustable capacitive response, allowing for precise and dependable pressure sensing over a broad operating range.

### 6.4 Mechanical sensitivity of the proposed rectangular CPS

The relationship between diaphragm thickness and dw/dP is seen in Figure 5f. dw/dP drops with increasing diaphragm thickness since of less deflection. When the thickness is greater than 20 μ*m*, the sensitivity significantly decreases for the given design parameters (Table 1). Therefore, 20 μ*m* is the ideal limit for the diaphragm thickness for this proposed sensor design.

## 7 Diaphragm deflection analysis under applied pressure using FEA simulation

Using FEA, the diaphragm deflection profile, normal mode and touch mode deformation are shown in Figures 6–8. Figures 6a–c shows the geometry of the pressure sensor model and how the rectangular diaphragm bends when pressure is applied to it. The cross-section view shows that the diaphragm bends the most at its center and gradually flattens out toward the edges.

**Figure 6:**
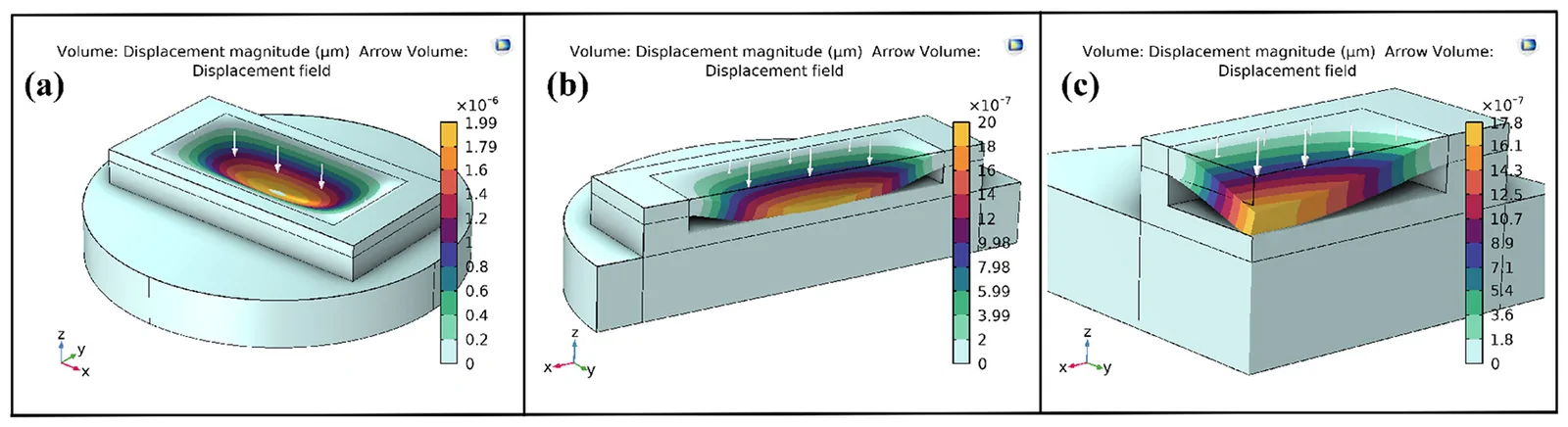
**(a)** Schematic structure of the proposed sensor design under applied pressure, **(b, c)** cross sectional views of internal deflection of diaphragm for proposed capacitive pressure sensor in FEA.

**Figure 7:**
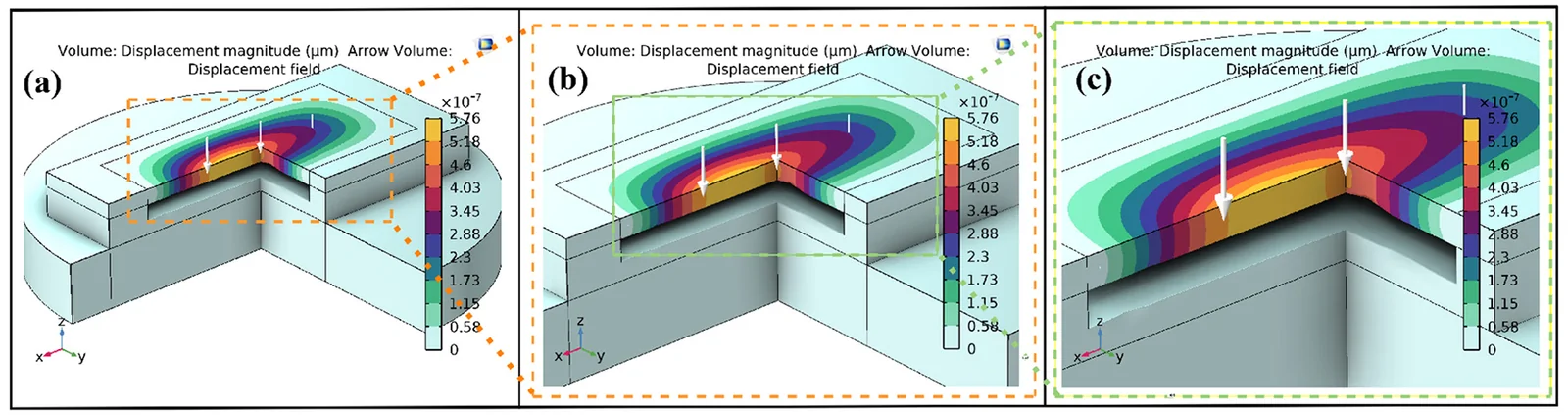
**(a)** Schematic cross sectional view of normal mode proposed capacitive pressure sensors, **(b, c)** internal cross sectional view of normal mode proposed capacitive pressure sensor in FEA.

**Figure 8:**
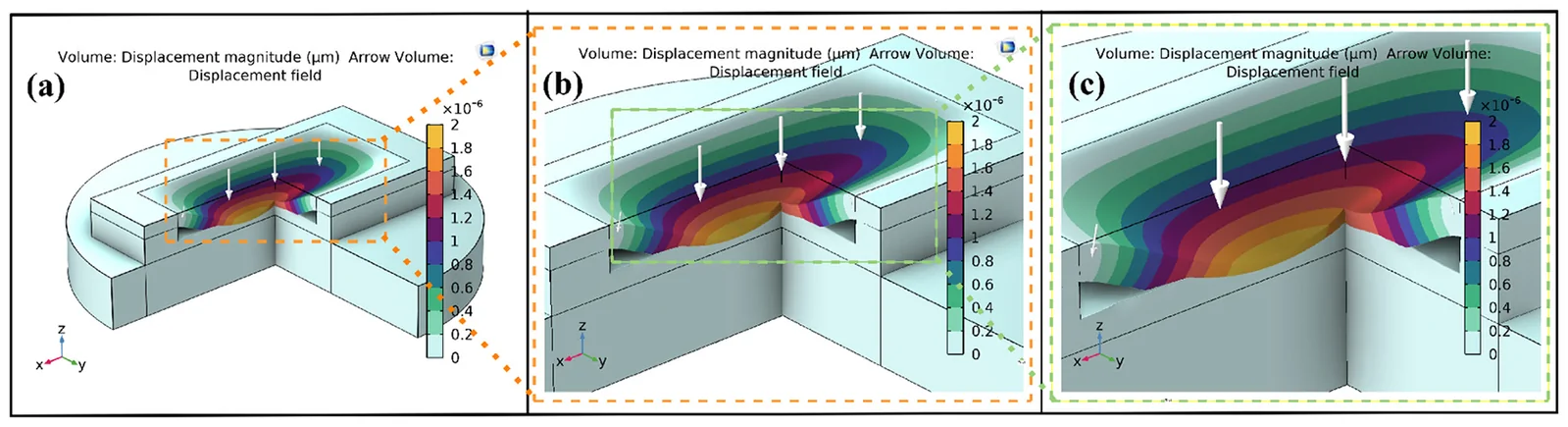
**(a)** Schematic cross view of touch mode deflection distribution for proposed capacitive pressure sensor, **(b, c)** internal cross sectional views of touch mode deflection for proposed capacitive pressure sensors in FEA.

### 7.1 Diaphragm deflection in normal mode for the proposed rectangular CPS

When pressure is applied in the normal mode, the diaphragm bends downward without coming into contact with the bottom electrode. Depending on the boundary circumstances, the diaphragm center experiences the most deflection, which usually has a parabolic form. For minor deformations, the connection between applied pressure and deflection stays almost linear and as the distance between the electrode and the diaphragm gets smaller, the capacitance progressively rises. The FEA was used to carry out the simulation. FEA is an effective tool for precisely analyzing sensor performance and visualizing deformation. The diaphragm deflection is greatest in the middle and diminishes as it moves outward. A cross sectional view of the suggested sensor with the normal mode is shown in Figures 7a–c.

### 7.2 Diaphragm deflection in touch mode for the proposed rectangular CPS

In the touch mode, the middle part of the diaphragm makes contact with the bottom electrode as the pressure rises above a predetermined threshold. As a result, the diaphragm center flattens while the surrounding areas keep bending. With increasing pressure, the contact area grows and the deflection becomes nonlinear. Since the effective contact area grows so quickly, capacitance rises sharply as a result. In general, touch mode adds a mix of flat (contact) and curved (non-contact) areas, increasing the sensitivity of the sensor response. A cross-sectional view of the suggested sensor with the Touch mode is shown in Figures 8a–c.

## 8 Deep learning-based performance modeling of rectangular MEMS CPS

The earlier sections show that rectangular diaphragms give better sensitivity and linearity than circular and square ones. This was confirmed using both mathematical modeling and FEA simulation. These simulations check how shape, size and operating conditions affect performance. But doing this for every possible combination takes too much time and computing power. To solve this problem without losing accuracy, a deep neural network (DNN) surrogate model is used instead. Once trained on the existing simulation data, the DNN can predict all sensor outputs almost instantly, making it possible to explore the full design space quickly something that would be too slow to do with direct simulation alone. The analytical model provides physical insight into sensor behavior and supports preliminary validation. FEA delivers high-fidelity, simulation-based data with validation across the full design parameter space. The DNN provides the computational efficiency required for design optimization. Since each method offers a capability the other two lack, all three are essential and complementary components of the overall framework. The proposed sensor design shows strong performance but conventional design relies heavily on running numerous FEA simulations. These simulations evaluate variations in shape, size and operating conditions. This process becomes computationally expensive and time-consuming. A machine learning based surrogate model (DNN) is used to overcome this limitation. The Model is trained on existing simulation data to learn the relationship between design parameters and performance characteristics. Once trained, it can predict sensor performance rapidly and accurately. This eliminates the need for new simulations at every design iteration, significantly reducing computational cost.

A two-level evaluation framework is employed, holding out 10% of data as a sealed test set while performing five-fold cross-validation on the remaining 90%, with fold-wise MinMax normalization to prevent leakage. Figure 9 shows the MLP model features four hidden layers of 20 neurons each with ReLU activation, trained via Adam optimiser and MSE loss in TensorFlow/Keras, supported by EarlyStopping and ReduceLROnPlateau for stable convergence. Final performance is reported using MAPE, *R*^2^, RMSE and MAE, computed in original physical units after inverse transformation to ensure meaningful and accurate evaluation.

**Figure 9:**
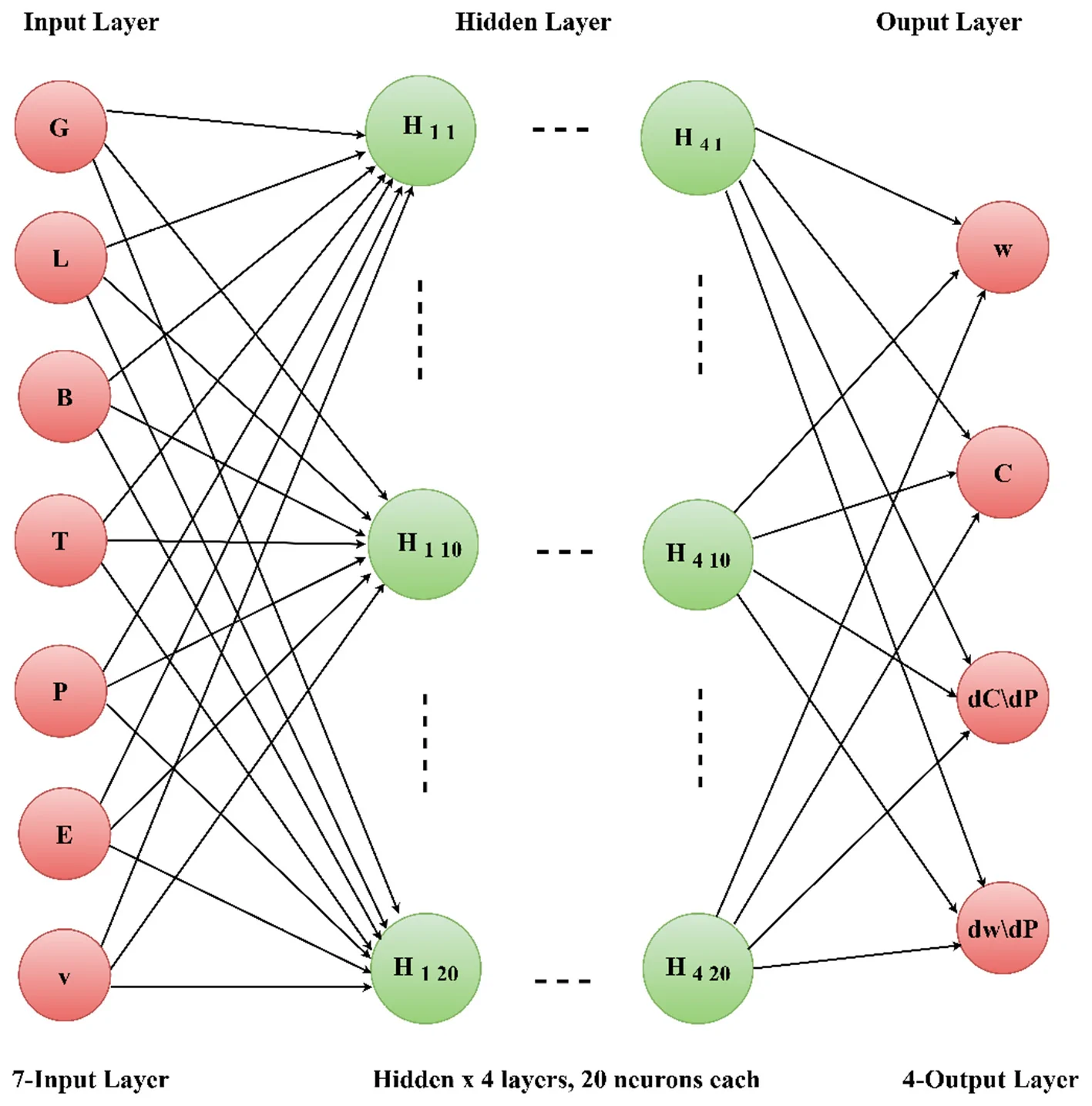
Multilayer perceptron (MLP) architecture for the FEA output of the proposed rectangular CPS.

### 8.1 Hyperparameter selection and network configuration

The network architecture was determined through a systematic grid search over the number of hidden layers (1, 2, 4, and 6) and the number of neurons per layer (20, 32, and 64). Each candidate configuration was assessed using five-fold cross validation on the development set and the configuration achieving the lowest mean validation MAPE together with the highest *R*^2^ was selected as optimal. The results of this search are summarized in Table 2. The selected architecture four hidden layers of 20 neurons each provided the best trade-off between model expressiveness and regularization given the 3,000 sample dataset. He-uniform initialization was adopted for the network weights, as it is specifically suited to ReLU activations, while the Adam optimizer with an initial learning rate of 0.001 was used to train the surrogate model. Figure 10 shows the training and validation loss and MAE curves. Both curves drop smoothly and stay close to each other throughout training. The validation curve does not rise while the training curve keeps falling and neither curve gets stuck at a high error value. This shows that the model is neither overfitting nor underfitting and that it generalizes well to new, unseen data.

**Table 2:** Hyperparameter grid search results (five-fold CV, development set).

Configuration	Val MAPE	Val *R*^2^	Convergence	Generalization	Decision
1 hidden layer (20)	0.51%	0.9943	Under-fit	Poor	Insufficient capacity
2 hidden layers (20)	0.38%	0.9971	Slight under-fit	Fair	Not enough depth
4 hidden layers (20) ✓	0.22%	0.9993	Converged	Excellent	Selected architecture
4 hidden layers (32)	0.28%	0.9985	Slight overfit	Good	Extra parameters unnecessary
4 hidden layers (64)	0.31%	0.9981	Overfit	Good	Overfit on 3K samples
6 hidden layers (20)	0.24%	0.9991	Converged	Good	Marginal gain, slower

**Figure 10:**
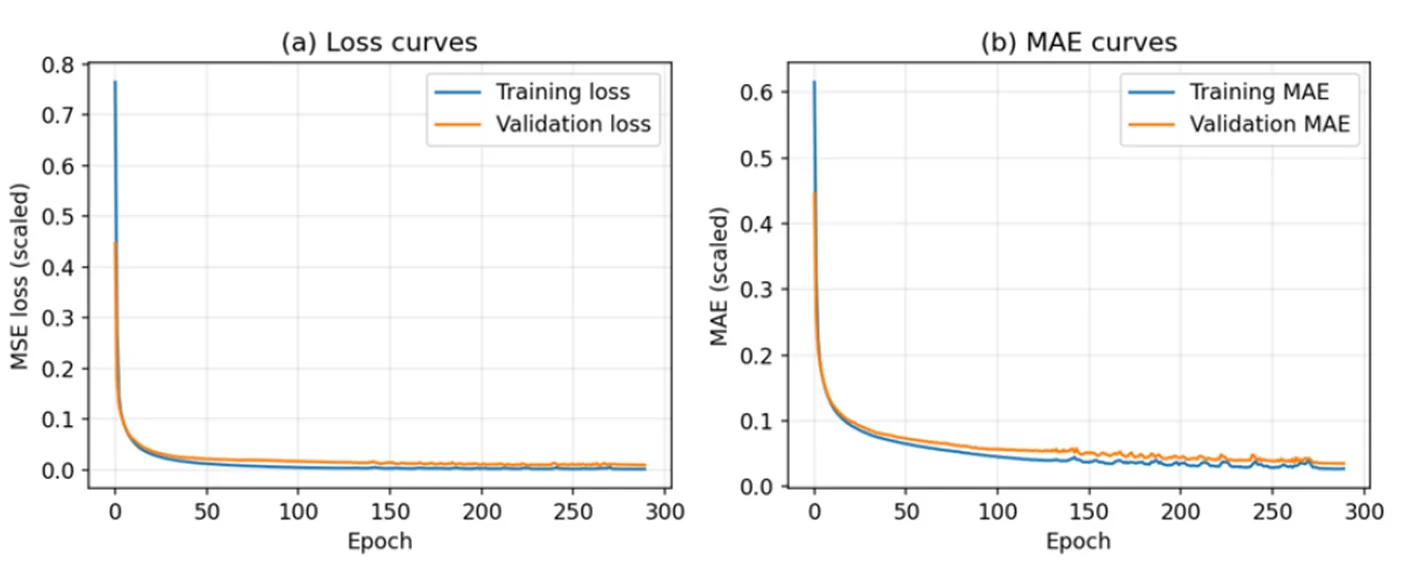
Training vs. validation **(a)** MSE loss and **(b)** MAE across epochs for the final 4-layer × 20-neuron DNN. The two curves remain close throughout training with no divergence, indicating neither overfitting nor underfitting.

### 8.2 Deep learning surrogate model for the proposed MEMS CPS

A deep neural network (DNN) was developed to predict the characteristics of MEMS capacitive pressure sensors as a fast surrogate alternative to computationally expensive FEA simulations. The model demonstrated excellent predictive accuracy and stability, with five-fold cross-validation yielding MAPE values below 0.26% and *R*^2^ values greater than 0.998 across all output variables. Smooth convergence of training and validation losses, with no significant overfitting, was achieved through the effective use of EarlyStopping and adaptive learning-rate reduction techniques.

### 8.3 Visual and quantitative assessment of regression performance using predicted vs. actual scatter plots

The predicted vs. actual scatter plots presented in Figures 11a–d provide a direct visual assessment of the model's regression accuracy across all four output variables of the MEMS capacitive pressure sensor. In each subplot, the predicted values are plotted against their corresponding actual values from the held-out test set, with a black dashed diagonal representing the ideal one-to-one correspondence.The data points cluster tightly along this ideal line for all four outputs deflection, capacitance, dC/dP, and dw/dP confirming that the trained DNN accurately captures the linear relationships between the seven input design parameters and the sensor output characteristics. The quantitative metrics displayed in each subplot, with *R*^2^ values exceeding 0.999 and MAPE below 0.22%, further reinforce the visual evidence of high predictive fidelity across the full operating range of the sensor design space.

**Figure 11:**
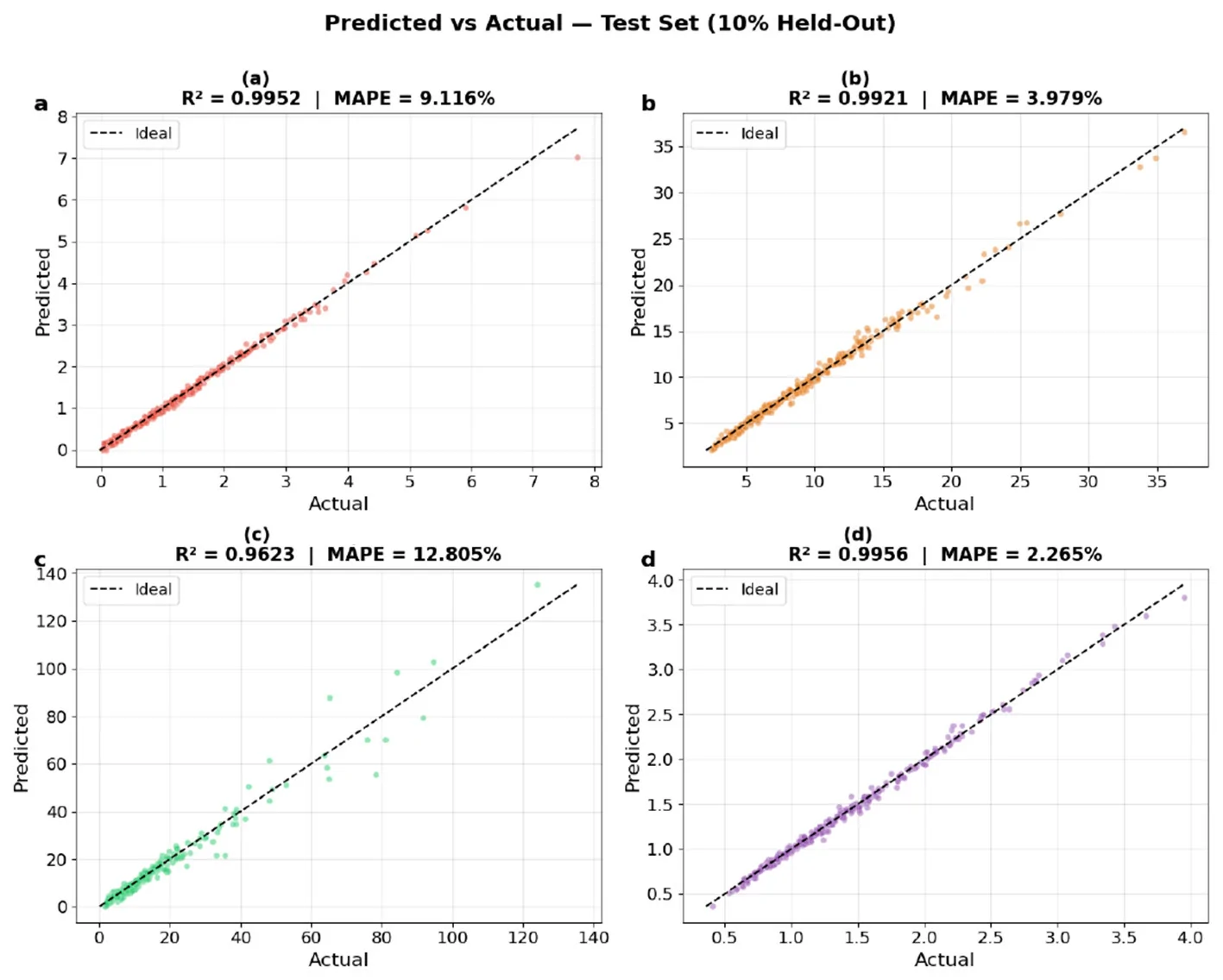
Regression accuracy assessment via predicted vs. actual plots. **(a)** Deflection (um). **(b)** Capacitance (pF). **(c)** dC/dP (pF/MPa). **(d)** dw/dP (um/MPa).

### 8.4 Point-wise absolute error analysis for assessing prediction consistency and stability of the proposed surrogate model

The per-sample absolute prediction error plots presented in Figures 12a–d illustrate the point wise error behavior of the trained model across all 300 samples of the held-out test set. For each output variable, the absolute difference between the actual and predicted values is plotted against the test sample index, with a black dashed horizontal line marking the mean absolute error.

**Figure 12:**
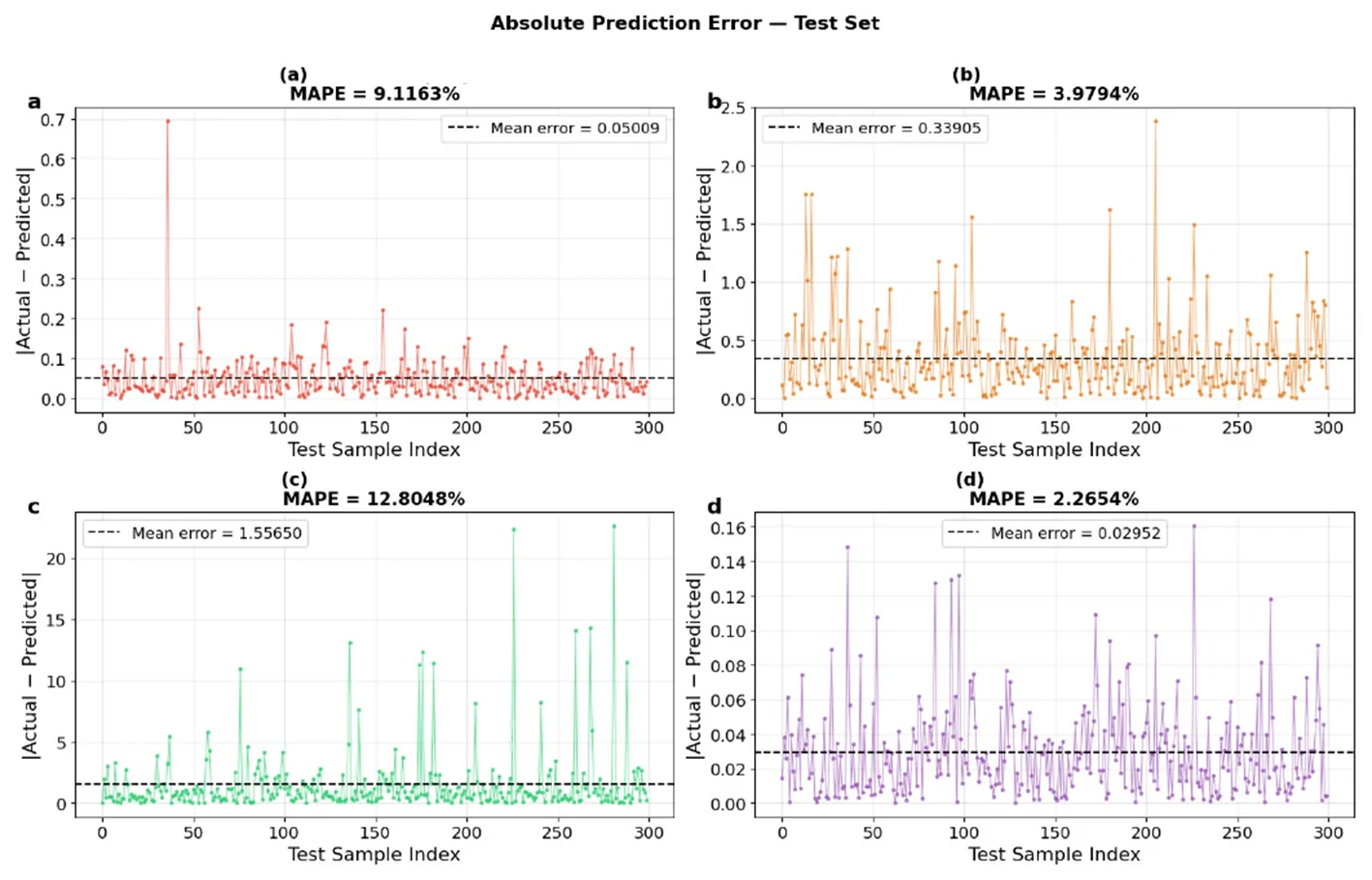
Point-wise error evaluation across the hold-out test samples. **(a)** Deflection (um). **(b)** Capacitance (pF). **(c)** dC/dP (pF/MPa). **(d)** dw/dP (um/MPa).

The error profiles remain consistently low and stable across all samples for Deflection, Capacitance and dw/dP, with only minor isolated spikes observed at boundary regions of the design space where training data density is relatively lower. The dC/dP output exhibits slightly higher individual error magnitudes, attributable to its exceptionally wide dynamic range. Nonetheless, the mean error line remains close to zero in all cases, confirming uniform and stable generalization across the entire test set.

### 8.5 Residual distribution histograms for quantifying prediction bias, error symmetry and statistical reliability of the proposed MLP-based surrogate model

The residual distribution histograms presented in Figures 13A–D provide a statistical assessment of prediction bias and error symmetry for the trained DNN model evaluated on the 300-sample held-out test set. For each output variable, residuals are computed as the signed difference between actual and predicted values and visualized as histograms with 35 bins, accompanied by a black dashed vertical line at zero representing ideal unbiased prediction and a red dotted line indicating the actual mean residual. Across all four output variables, the residual distributions exhibit approximately symmetric, bell-shaped profiles centered close to zero, confirming that the model does not exhibit systematic over-prediction or under-prediction tendencies. The narrow spread of the distributions for capacitance and dC/dP reflects their lower prediction variance, while the slightly broader distribution observed for dw/dP is consistent with the challenges imposed by its wide dynamic range. Overall, the residual analysis confirms that the prediction errors are random and normally distributed, satisfying a key statistical requirement for reliable and unbiased regression modeling.

**Figure 13:**
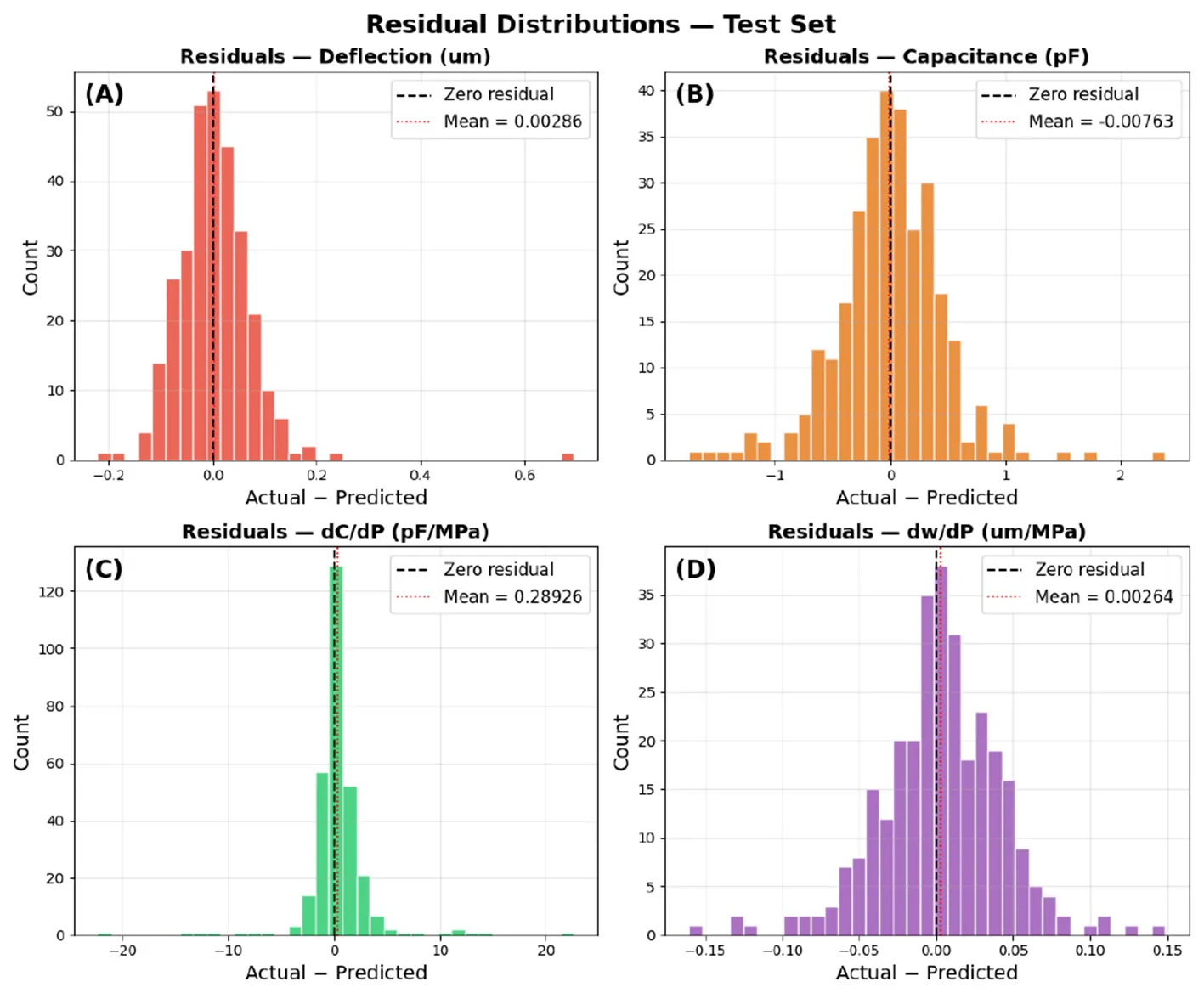
Statistical assessment of prediction bias via residual distributions; **(A)** residual distribution for deflection **(B)** residual distribution for capacitance (C) residual distribution for capacitive sensitivity (dC/dP) and **(D)** residual distribution for mechanical sensitivity (dw/dP).

## 9 Comparison with related works

From the Table 3, diaphragm geometry significantly influences the sensitivity of touch-mode capacitive pressure sensors, with rectangular diaphragms achieving the highest sensitivity (28.8123 pF) due to their directional deflection and rapid contact area expansion along the longer axis, outperforming square (4.5 pF) and circular (7.8 pF at 1.8 MPa) geometries. While prior works (Mishra et al., 2012; Jakati et al., 2016; Lobur et al., 2016; Zhang et al., 2019) relied exclusively on FEA to analyse such sensor characteristics without any machine learning component, Park et al. (2021) and Chen and Chen (2022) demonstrated the viability of DNN FEA surrogate models, with Chen and Chen (2022) predicting two sensor outputs from four inputs and achieving MAPE below 0.30% on a single random split. The proposed model improves on earlier work in three main ways. It uses a wider range of design parameters as inputs, predicts more output targets and uses a stronger testing method based on five-fold cross validation with a separate hold-out test set. Together, these extensions result in a more comprehensive and statistically rigorous surrogate model, achieving an MAPE of 0.22 % and an *R*^2^ of 0.9993 outperforming the existing benchmark while covering a broader and more practically relevant MEMS design space. Table 4 lists the benchmarking results of the proposed MLP against standard regression algorithms.

**Table 3:** A Comparative Evaluation of Existing Machine Learning Models and a Proposed DNN Framework for MEMS Behavioral Prediction.

References	Method	Capacitance	Inputs	Outputs	MAPE (%)	*R* ^2^
Mishra et al. (2012)	FEA only	–	4	–	–	–
Jakati et al. (2016)	FEA comparative	–	3	–	–	–
Zhang et al. (2019)	FEA + analytical	–	4	–	–	–
Kang et al. (2017)	FEA + analytical	4.5 pF	4	–	–	–
Kang et al. (2021)	FEA + numerical computation	7.8 pF	4	–	–	–
Park et al. (2021)	DNN + FEA	–	1	1	< 0.50	0.998
Chen and Chen (2022)	MLP + FEA	–	4	2	< 0.30	N/A
**Proposed work**	**MLP + five-fold CV**	**28.8123 pF**	**7**	**4**	**< 0.22**	**>0.999**

Bold values indicate the best performance among the compared methods/results.

**Table 4:** Benchmarking of proposed MLP against standard regression algorithms (hold-out test set, 300 samples).

Model	MAPE (%)	R^2^	Inference
**MLP (proposed)**	0.22%	>0.999	< 10 s
CNN (1D)	1.85%	0.9912	< 5 s
LSTM	3.24%	0.9847	< 8 s
XGBoost	4.51%	0.9723	< 1 s
SVR (RBF)	7.89%	0.9481	< 2 s
Random Forest	3.12%	0.9819	< 1 s

Bold values indicate the best performance among the compared methods/results.

## 10 Conclusion

It is clear from the results, that rectangular diaphragms provide more benefits in terms of performance and design freedom when compared to circular and square diaphragms for CPS. The design improves linearity by allowing for a smoother and more consistent capacitance variation with pressure. Conduct and non-conduct modes can be switched smoothly by adjusting the diaphragm's aspect ratio. The rectangular diaphragm design of the suggested sensor is responsible for its noticeably increased sensitivity. The benefits of employing rectangular diaphragms are confirmed by analytical and modeling results, which emphasize enhanced sensitivity, capacitance and mechanical durability. Most notably, the trained DNN delivers predictions dramatically faster than traditional FEA simulations significantly reducing computational cost. This combination of high accuracy and computational efficiency makes the proposed surrogate model highly effective for rapid design-space exploration, optimization and sensitivity analysis of MEMS capacitive pressure sensors.

## Data Availability

The original contributions presented in the study are included in the article/supplementary material, further inquiries can be directed to the corresponding author.
